# Frontline Blinatumomab in Older Adults with Philadelphia Chromosome-Negative B-Cell Acute Lymphoblastic Leukemia

**DOI:** 10.3390/ph13060124

**Published:** 2020-06-16

**Authors:** Sandrine Niyongere, Gabriela Sanchez-Petitto, Jack Masur, Maria R. Baer, Vu H. Duong, Ashkan Emadi

**Affiliations:** 1 Departments of Medicine, University of Maryland School of Medicine, Baltimore, MD 21201, USA; Sandrine.Niyongere@umm.edu (S.N.); Gabriela.Sanchez@umm.edu (G.S.-P.); jmasur@som.umaryland.edu (J.M.); mbaer@umm.edu (M.R.B.); vduong@umm.edu (V.H.D.); 2 Marlene and Stewart Greenebaum Comprehensive Cancer Center, Baltimore, MD 21201, USA; 3 Departments of Pharmacology, University of Maryland School of Medicine, Baltimore, MD 21201, USA

**Keywords:** blinatumomab, acute lymphoblastic leukemia (ALL), elderly

## Abstract

Outcomes of acute lymphoblastic leukemia (ALL) in older adults treated with chemotherapy are poor. The CD19/CD3 bispecific T-cell engager (BiTE) antibody blinatumomab is approved for refractory, relapsed or minimal/measurable residual disease (MRD)-positive B-cell ALL, but there is little experience in the upfront setting, including in older patients. We retrospectively analyzed outcomes of blinatumomab monotherapy in five newly diagnosed Philadelphia chromosome-negative B-cell ALL patients over 70 years. Three had cytokine release syndrome, treated with dexamethasone and/or tocilizumab, and four patients had neurotoxicity, treated with dexamethasone, without blinatumomab interruption. All five achieved complete remission (CR) after cycle one, three with undetectable MRD. All five were alive at 8 to 15 months. Three remained in MRD-negative CR. Two relapsed after cycle 3, one with extramedullary disease. In our small cohort of patients over 70 years, blinatumomab was safe initial therapy and produced a high response rate.

## 1. Introduction

Despite recent advances, outcomes in older adults with newly diagnosed acute lymphoblastic leukemia (ALL) remain poor, with higher early mortality, lower complete remission (CR) rates, higher relapse rates and shorter survival than younger patients [1]. Reasons include both the poor tolerance of intensive chemotherapy and the higher incidence of leukemia-related poor-risk features, such as adverse karyotype, with increasing age, so that intensive therapies, even if tolerated, are less likely to be effective [1]. Older patients with Philadelphia chromosome (Ph)-positive ALL may tolerate and respond to tyrosine kinase inhibitor-based therapy [2], but older patients with Ph-negative ALL lack tolerable and effective treatment options. Additionally, older adults with ALL are often excluded from clinical trials, with enrollment often limited to patients less than age of 60 or 65 years of age [3,4]. There is therefore a lack not only of effective therapeutic options, but also of evidence-based guidelines for treatment of ALL in patients 70 years of age and older. 

Given that older ALL patients tolerate chemotherapy poorly and also frequently have chemoresistant disease, non-chemotherapy-based treatment approaches are attractive. Blinatumomab is a CD19/CD3 bispecific T-cell engager (BiTE) antibody that is currently approved by the United States Food and Drug Administration (FDA) and the European Medical Agency (EMA) for use in adults and children with refractory or relapsed B-cell ALL or with B-ALL in first or second CR with minimal/measurable residual disease (MRD) detected as 0.1% or greater [5,6,7]. However, there is little experience with blinatumomab in the upfront setting, including in older patients. A phase II Southwest Oncology Group (SWOG) clinical trial of blinatumomab in 29 B-ALL patients 65 years and older published in abstract form [8] demonstrated good tolerability and a favorable response rate. Here, we report five patients over age 70 years receiving single-agent blinatumomab as initial therapy for Ph-negative B-ALL. 

## 2. Results

Five patients over age 70 years with newly diagnosed Ph-negative B-lineage ALL received blinatumomab monotherapy (Table 1). Ages ranged from 71 to 86 years. Comorbidities included but were not limited to congestive heart failure (CHF), coronary artery disease and atrial fibrillation. ALL was therapy-related in two patients, following treatment for multiple myeloma and for prostate cancer. Median white blood cell (WBC) count was 11,800 per microliter (/mcL), absolute neutrophil count (ANC) 1330/mcL, hemoglobin 9.0 g/dL, and platelet 73,200/mcL. Percentage of marrow lymphoblasts ranged from 31 to 84. CD19 was expressed on over 70% lymphoblasts by flow cytometry in all. All five patients had CD22 expression with three of five patients having over 90% CD22 expression on lymphoblasts while two patients had between 50 and 70% CD22 expression. Cytogenetic analysis demonstrated normal karyotypes in two patients, hyperdiploidy in two, and a complex karyotype in one (Table 1). One patient had lymphoblasts in the cerebrospinal fluid (CSF) at diagnosis.

All patients received continuous infusions of blinatumomab for 4 weeks, per the approved dose and schedule [9]. Three patients had symptoms of cytokine release syndrome (CRS), two grades 3 and one grade 2, all received dexamethasone and one received tocilizumab. Four patients had symptoms of neurotoxicity, successfully treated with dexamethasone. Blinatumomab infusions were not interrupted in any patient. Other adverse events (AEs) included fatigue (four patients), neutropenia (three patients), anemia (three patients), fever (two patients) and headache (two patients). 

All patients received prophylactic intrathecal (IT) chemotherapy, including cytarabine, methotrexate and hydrocortisone. The patient with lymphoblasts in the CSF at diagnosis received three additional doses of IT chemotherapy, until clearance of blasts. All patients received pre-treatment dexamethasone before each cycle of blinatumomab. 

All patients underwent bone marrow aspirate and biopsy on Day 28 ± 3 of Cycle 1, the last day of blinatumomab infusion. All five patients achieved complete hematologic remission at the end of Cycle 1, with no morphologic evidence of ALL. MRD was not detected in 3 patients at the end of Cycle 1. Gökbuget and colleagues demonstrated the importance of achieving undetectable MRD as a prognostic factor in patients treated with blinatumomab. In a phase II clinical trial NCT01466179 evaluating MRD response with blinatumomab in relapsed/refractory B-cell ALL, the study observed improved longer duration of response and relapsed free survival in the patients who achieved undetectable MRD with blinatumomab [10]. 

One patient received 6 cycles of blinatumomab, one patient received 4 cycles and three patients received 3 cycles with prophylactic IT chemotherapy. All five patients are alive with follow-up ranging from 8 to 15 months. Three are in ongoing MRD-negative CR. Two patients relapsed after cycle 3 of blinatumomab, one with new extramedullary disease (cervical and mediastinal lymphadenopathy). Both patients who relapsed did not maintain CD19 expression on lymphoblasts. Of note, the patient with CSF lymphoblasts at time of diagnosis has not had a CSF recurrence. The two patients who relapsed have started the anti-CD22 immunoconjugate inotuzumab as second-line therapy. 

## 3. Discussion

In our small cohort of patients over 70 years of age, we observed that single-agent blinatumomab could be administered safely and produced a high response rate. 

Chemotherapy outcomes for older adults with ALL remain poor, leading to increased interest and research focusing on chemotherapy-free strategies. The SWOG conducted a phase II trial of blinatumomab followed by prednisone, vincristine, methotrexate, and 6-mercaptopurine (POMP) maintenance chemotherapy in older patients with newly diagnosed Ph-negative B-cell ALL, reported in abstract form [8]. Twenty-nine patients 65 years and older received 1–2 cycles of blinatumomab as induction therapy, then 3 cycles of blinatumomab as post-remission therapy, followed by POMP maintenance chemotherapy for 18 months. Median age was 75 years (range, 66–84 years). The response rate was 66%, with only one patient requiring two cycles for response, and 12 of 17 patients tested were MRD-negative. Treatment was generally well tolerated, with a single induction death. Estimated one-year disease-free survival was 55%. Our series augments this experience, also demonstrating safety and efficacy. CRS and neurotoxicity remain important complications of blinatumomab treatment. In our small cohort they could be managed and did not require treatment interruption.

Advani and colleagues presented very compelling data from a SWOG 1318 study, which was a phase II trial of blinatumomab followed by POMP maintenance in elderly patients with newly diagnosed Ph-negative B-cell ALL [8]. The primary objective of the study was to estimate the three-year overall survival (OS) and there were several similarities between our study. Both our study and the SWOG study administered IT chemotherapy for central nervous system (CNS) prophylaxis and we both evaluated MRD status at the end of induction cycle one [8]. Both our study and the SWOG study assessed response at the completion of 1–2 cycles of blinatumomab and graded toxicities according to National Cancer Institute (NCI) Common Terminology Criteria for Adverse Events (CTCAE). The difference between our study and the SWOG study is that the eligibility age for the SWOG study was 65 years of age and older while all of our patients were age 70 years or older. The SWOG study excluded patients with evidence of CNS disease and our study included patients with CNS involvement (one of our patients had positive CNS involvement by CSF analysis which cleared with treatment with IT chemotherapy). The SWOG study had longer follow up compared to our study that focused on safety and tolerability during the initial cycles of blinatumomab therapy. We both observed that blinatumomab is not only well-tolerated but also effective in the treatment of newly diagnosed Ph-negative B-cell ALL in elderly patients, however, additional research is required to identify the durability of the response observed.

Multi-agent regimens including blinatumomab are also being tested in older ALL patients. Mini-hyper-CVD (hyperfractionated cyclophosphamide, vincristine, dexamethasone) low-intensity chemotherapy with inotuzumab ozogamicin, with or without blinatumomab, improved 3-year event-free and OS rates, compared to standard hyper-CVAD alone, in newly diagnosed Ph-negative B-ALL patients older than 60 years based on a propensity score analysis [11]. Sequential therapy with inotuzumab ozogamicin followed by blinatumomab is also being studied in a current cooperative group clinical trial.

Blinatumomab in the frontline setting appears to be well tolerated, safe and effective in older adults with B-lineage ALL. Durability of responses may be augmented by combination regimens.

## 4. Methods

We conducted a retrospective analysis of outcome of blinatumomab monotherapy for newly diagnosed Ph-negative B-cell ALL based on 2016 World Health Organization (WHO) criteria [12] in five patients older than 70 years at the University of Maryland Greenebaum Comprehensive Cancer Center, outside of a clinical trial. A waiver of Health Insurance Portability and Accountability Act (HIPAA) authorization for release of the Protected Health Information (PHI) identified in the research application was reviewed and approved for this study by University of Maryland Baltimore (UMB) Institutional Review Board (IRB). The study was conducted in accordance with the Declaration of Helsinki, and the protocol was approved by the University of Maryland School of Medicine IRB (Project identification code: GCCC19138).

Blinatumomab was administered per the approved dose and schedule [9].

CR was defined by red blood cell transfusion independence, ANC >1000/mcL and platelet count >100,000/mcL and less than 5% bone marrow blasts. CR with absence of MRD was defined as CR with absence of abnormal lymphoblasts by flow cytometric analysis performed by Hematologics, Inc. (Seattle, WA, USA) (estimated lower level of detection, 0.02%) [13]. Relapse was defined by presence of >5% lymphoblasts in the bone marrow or peripheral blood or pathologic evidence of extramedullary ALL. Duration of response (DOR) was defined as interval from CR to relapse or death from any cause, and OS as interval from ALL diagnosis to death from any cause. Date of last follow-up was 1 May 2020.

AEs, including neurotoxicity and CRS, were documented and graded per NCI CTCAE Version 5.0.

## Figures and Tables

**Table 1:** Patient and Disease Characteristics.

Patient	1	2	3	4	5
**Age (years)**	86	76	85	71	76
**Gender**	F	F	M	M	M
**Comorbidities**	Atrial fibrillation s/p ablation, MI, CHF	Multiple myeloma	Atrial fibrillation	Glaucoma	Prostate cancer, atrial fibrillation, MI × 3 with 5 stents
**WBC (× 10^9^/L)**	38	2.1	2.1	1.4	15.6
**Blood blasts (%)**	77	0	0	2	64
**Hemoglobin (g/dL)**	8.2	12.3	7.4	10.3	8.2
**Platelets (× 10^9^/L)**	22	104	186	63	14
**BM blasts (%)**	-*	83	35	31	84
**CD19 expression (%)**	99	88	71	79	93
**Karyotype**	46,XX,add(6)(q27),del(12)(p12)[3]/55,XX,+3,+5,+8, +11,del(12),+del(12),+17, +18,+21,+22 [17][cp20]	46,XX [cp23]	59-60, Hyperdiploid [2]/46,XY [18][cp20]	46,XY [cp20]	46,XY,add(1)(q43),+5,t(5;10)(q11.2;p11.2),der(7)t(7;18)(q10;q10),del(9)(p13),t(15;20)(q11.2;q11.2),-18 [9]/46,XY [11][cp20]
**CSF involvement**	Yes	No	No	No	No
**CR post-induction**	Yes	Yes	Yes	Yes	Yes
**MRD post-induction (%)**	0.06%	negative	negative	0.3%	negative
**CR duration (months)**	3	3	10.5+	6+	3.5+
**CRS**	No	Grade 2	Grade 3	Grade 3	No
**Tocilizumab use**	No	No	No	Yes	No
**Neurotoxicity**	Yes	Yes	Yes	Yes	No
**Steroid use**	Yes	Yes	Yes	Yes	No
**Blinatumomab cycles**	3	3	4	6	3
**Relapse**	Yes (extramedullary)	Yes	No	No	No
**Survival (months)**	10+	10+	15+	11+	8+

F = female; M = male; s/p = status post; MI = myocardial infarction; CHF = congestive heart failure; WBC = white blood cell count; BM = bone marrow; cp is the composite karyotype, which contains all clonally recurring abnormalities in setting of karyotypic heterogeneity; CSF = cerebrospinal fluid; CR = complete remission; MRD = minimal residual disease; CRS = cytokine release syndrome; L = liter. * Bone marrow biopsy was not performed at the time of diagnosis, ALL was diagnosed with peripheral blood flow cytometry. The numbers in square brackets are not references; these numbers in cytogenetic reports are the total number of cells in which the clonal changes were observed.
